# Design of Novel Knot-like Structures Based on Ovotransferrin Fibril–Gum Arabic Complexes: Effective Strategies to Stabilize Pickering Emulsions

**DOI:** 10.3390/foods12203767

**Published:** 2023-10-13

**Authors:** Zihao Wei, Yue Dong, Xiaolong Li, Meng Wang, Keming Zhang

**Affiliations:** State Key Laboratory of Marine Food Processing & Safety Control, College of Food Science and Engineering, Ocean University of China, Qingdao 266404, China

**Keywords:** ovotransferrin fibril, gum arabic, knot-like structure, interfacial property, oleogel-based Pickering emulsion, stability

## Abstract

This work aimed to clarify the effects of gum arabic (GA) on the morphology and properties of ovotransferrin fibrils (OVTFs). By constructing OVTF–GA complexes and exploring the dispersion stability, turbidity and the ζ-potential of the complexes, the optimum mass ratio of OVTFs to GA and pH for complex formation were confirmed as being 1:1 and pH 4.6, respectively. The interaction between OVTFs and GA was determined to be predominantly driven by electrostatic attraction. The OVTF–GA complexes exhibited a knot-like structure when observed using atomic force microscopy. Then, OVTFs and OVTF–GA complexes were compared in terms of contact angle, surface hydrophobicity and dynamic interfacial tension. The combination of OVTFs and GA decreased the contact angle of OVTFs from 80.85° to 70.36°. In comparison with OVTFs, OVTF–GA complexes reduced the oil–water interfacial tension to a lower level (8.14 mN/m). Furthermore, the capacities of OVTF–GA complexes in stabilizing emulsions were explored. OVTF–GA complex-stabilized oleogel-based Pickering emulsion (OGPE) was constructed, and OVTF-stabilized oleogel-based Pickering emulsion (OPE) was used as the control. OGPE had a higher emulsified phase volume fraction (EPVF) and stability index (SI). The EPVF of OGPE was 100.0% and 99.4% before and after one-month storage, respectively, compared with 98.3% and 95.7% of OPE. This work can provide some useful references for the design of biopolymers with novel structures composed of protein fibrils and polysaccharides, which may also help to construct and apply protein fibril–polysaccharide complexes under specific needs.

## 1. Introduction

Food is a complex multi-component system, and the interactions between different components cannot be ignored. Proteins and polysaccharides are the most dominant components in food, and protein–polysaccharide interactions have significant impacts on the properties of food, such as texture, flavor, and appearance [1]. Complexation of proteins and polysaccharides can be driven by electrostatic interaction, hydrogen bond, enzymatic crosslinking, Maillard reaction and so on, which will influence the functional characteristics of proteins, including gelation properties, film-forming properties, emulsifying properties and so on [2,3,4]. The physicochemical properties of protein–polysaccharide complexes are strongly influenced by protein conformation, which is impressionable to external environments such as pH and temperature. The acid–heat treatment of proteins is a well-known method to fabricate fibrils. The high aspect ratio of protein fibrils endows them with some special characteristics, such as excellent emulsibility, high viscosity, and outstanding gelation properties [5]. Therefore, protein fibrillation is considered a powerful way to expand and improve protein function. It is widely known that the field of protein–polysaccharide interaction has been basically established. However, information on the interactions between protein fibrils and polysaccharides is relatively limited. It is reported that the increase in protein charges caused by heat-induced fibrillation may contribute to protein–polysaccharide complexation [6]. Hence, it is necessary to study the properties of protein fibril–polysaccharide complexes, which are beneficial to the construction of novel structures with desirable properties and functional value-added components for the food industry [7,8,9].

From the limited information available, research on protein fibril–polysaccharide interactions has focused on fibrils self-assembled from non-transferrin proteins, such as soy-protein-isolate fibrils, whey-protein-isolate fibrils, β-lactoglobulin fibrils and so on [10,11,12]. The interactions between fibrils transformed by transferrin and polysaccharides have not received sufficient attention. Since fibrils formed by different proteins may have different structures and functions, it is important to illustrate the fibrils formed by transferrin to meet specific requirements in foods. Ovotransferrin (OVT), as the iron-binding monomer glycoprotein, has high nutritional value, and its incorporation into food is considered an effective method for iron supplementation [13]. Moreover, OVT has some health benefits, such as antibacterial, antioxidant, anticancer and immunomodulatory effects [14,15]. Ovotransferrin fibrils (OVTFs) are short and flexible [13], and the influence of citrus pectin (CP) on OVTFs was clarified in our previous study. Under optimal preparation conditions, pearl chain-like OVTF–CP complexes were obtained via electrostatic interaction. On this basis, an oleogel-based Pickering emulsion stabilized by OVTF–CP complexes was constructed, and the pearl-chain structured complexes showed excellent advantages in constructing the delivery system [16].

Interestingly, the structures and properties of the protein fibril–polysaccharide complexes seem to depend on the properties of polysaccharides [17]. For example, β-lactoglobulin fibrils and sulfated κ-carrageenan were driven by electrostatic interactions to form necklace-like complexes [18]. In contrast, electrostatic attraction between β-lactoglobulin fibrils and pectin gave rise to nanotapes [10]. Therefore, the interaction of protein fibrils and polysaccharides with different structures may produce various functional value-added components that can meet the different needs of the food industry. Gum arabic (GA), as the emulsifier and thickener, is derived from the trunk secretions of the acacia genus in the legume family and is widely used in food industries [19,20]. Arabinogalactan fraction (AG), arabinogalactan–protein complex fraction (AGP) and glycoprotein fraction (GP) are the main components of GA [21]. Compared to CP, GA has a better emulsifying capacity due to the presence of AGP, where hydrophobic polypeptides are adsorbed at the oil–water interface and hydrophilic carbohydrates extend into the water phase [22]. In addition, GA exhibits outstanding stability at a wide range of pH values, high ionic strengths and high temperature, which is often used as a natural emulsifier, stabilizer, thickener, and film-former [23,24]. Compared to CP and other polysaccharides, GA has a lower viscosity, higher water solubility and lower pKa value [25]. Therefore, an in-depth exploration of protein fibril–GA complexes is beneficial to provide natural emulsifiers with excellent emulsification properties for the food industry.

This paper aims to explore the interactions between OVTFs and GA as well as the effect of GA on the emulsifying ability of OVTFs. Firstly, the optimal preparation conditions of OVTF–GA complexes were determined by analyzing their dispersion stability, turbidity and ζ-potential. Then, the interactions of OVTFs with GA and the effects of GA on the properties of OVTFs were investigated by comparing the properties of OVTFs and OVTF–GA complexes. Finally, the ability of OVTF–GA complexes to stabilize the emulsion was explored by preparing the oleogel-based Pickering emulsion. The information gained from this work can provide some guidance in designing biopolymers with new structures and regulating the formation and physicochemical properties of protein fibrils according to specific needs.

## 2. Materials and Methods

### 2.1. Materials

Ovotransferrin (OVT, purity > 88%) was provided by Neova Technologies Inc. (Abbotsford, BC, Canada). Gum arabic (GA) was obtained from Macklin Co., Ltd. (Shanghai, China). Canola oil and candelilla wax were obtained from Yihai Kerry Food Marketing Co., Ltd. (Shanghai, China) and Aladdin Biological Technology Co., Ltd. (Shanghai, China), respectively. Other reagents were analytical grade.

### 2.2. Preparation of Ovotransferrin Fibrils (OVTFs)

First, OVT powders were added to the water (pH 2.0, 100 mM NaCl) and stirred at room temperature (350 rpm, 4 h) to obtain the OVT solution. The OVT solution was magnetically stirred at 90 °C for 24 h to fabricate the protein fibrils. The fibril suspension was cooled to room temperature using an ice bath to stop the fibrosis. Then, the fibril suspension was dialyzed against water (pH 2.0, 4 °C) for 24 h to remove sodium chloride and obtain OVTF suspension.

### 2.3. Preparation of OVTF–GA Complexes

GA solution was obtained via adding an appropriate amount of GA to ultra-pure water and making GA dissolve in water after sufficient magnetic stirring. According to different mass ratios (r = 5:1, 2:1, 1:1) of OVTF/GA, GA solution was added into OVTF suspension and the mixed suspension was adjusted to appropriate pH values (2.0–7.5) using HCl and NaOH with different concentrations (0.1 M, 1 M and 5 M) to acquire OVTF–GA complex suspensions by magnetic stirring (600 rpm, 6.5 h).

### 2.4. Turbidity

OVTF–GA complex suspensions with different OVTF/GA mass ratios (r = 5:1, 2:1, 1:1) and different pH values (2.0–7.5) were prepared and stood for 14 h or 9.5 h to observe the dispersion stability of suspensions. The turbidity of OVTF–GA complex suspensions after standing was measured using an UV-2355 UV-Vis spectrophotometer (Unico Instrument Co., Ltd., Shanghai, China) at 600 nm.

### 2.5. ζ-Potential

ζ-potential of OVTF–GA complexes with different OVTF/GA mass ratios (r = 5:1, 2:1, 1:1), OVTFs and GA at different pH (2.0–7.0) were examined using Nano ZS90 (Malvern Instruments, Worcestershire, UK).

### 2.6. Morphology Measurement

The morphology of different samples was investigated using atomic force microscopy (AFM, Benyuan Nano-Instruments., Ltd., Guangzhou, China). In this study, 10 μL of sample with a total polymer concentration of 10 μg/mL was dropped onto the mica sheet and dried. Then, the tap mode was selected for image capture using AFM.

### 2.7. Contact Angle

The contact angle (θ) of samples was tested using the optical contact angle meter (OCA 15EC, Dataphysics, Filderstadt, Germany). First, different samples were freeze-dried to powders. Then, the powdered sample was pressed into pieces and placed in a sample pool with canola oil. A syringe needle containing ultra-pure water was inserted into canola oil, and a 3 μL water droplet was injected into the surface of the sample tablet to obtain θ.

### 2.8. Surface Hydrophobicity

Firstly, the suspension was diluted into different protein concentrations (0.4, 0.2, 0.1, 0.05 and 0.025 mg/mL) using phosphate-buffered solution (PBS, 10 mM, pH 7.0). Subsequently, 1-anilinonaphthalene-8-sulfonic acid (ANS) solution (8 mM) was prepared using PBS (10 mM, pH 7.0). In this study, 20 μL of ANS solution was added to 4 mL of the sample, followed by 10 min of dark reaction. Finally, the fluorescence intensity was measured at 390 nm (excitation) and 470 nm (emission) using an F-4600 fluorescence spectrophotometer (Hitachi, Tokyo, Japan). The surface hydrophobicity was obtained by fitting the slope of the fluorescence intensity and sample concentration [26].

### 2.9. Dynamic Interfacial Tension

The optical contact angle meter (OCA 15EC, Dataphysics, Filderstadt, Germany) was used to determine the dynamic interfacial tension of samples at the oil–water interface by using the pendant drop method [27]. The sample was injected into the oil as a droplet. Image acquisition was conducted immediately, and the interfacial tension was calculated according to the L-Y equation.

(1) $$\Delta p=\gamma \left(\frac{1}{R_{1}}+\frac{1}{R_{2}}\right)$$

where ∆p denotes the pressure difference between the inside and outside of the liquid surface, γ is the interfacial tension, while R_1_ and R_2_ represent the curvature radii of the liquid surface.

### 2.10. Preparation of Oleogel-Based Pickering Emulsion

OVTF–GA complexes were used to stabilize oleogel-based Pickering emulsions to evaluate their emulsification property. To prepare oleogel composed of candelilla wax and canola oil, the additive amounts of candelilla wax were investigated. The specific operation referred to the method of Dong et al. [16]. Candelilla wax was added to canola oil with different mass fractions (1.0%, 1.1%, 1.2%, 1.3%, 1.4% and 1.5%, *w*/*w*) and heated at 80 °C for 5 min. The inverted method was used to judge the formation of oleogel. When vials carrying the sample could stay at the top of inverted vials at room temperature for more than 30 min, it suggested the successful formation of oleogel.

Through the above experiments, the gel concentration of candelilla wax was determined to be 1.1% (*w*/*w*). Oleogel (65% volume fraction) and OVTF–GA complex suspension were mixed and sheared at 10,000 rpm for 3 min using a T25 homogenizer (IKA-Werke GMBH & CO., Staufen, Germany) to obtain OVTF–GA complex-stabilized oleogel-based Pickering emulsion (OGPE). OVTF-stabilized oleogel-based Pickering emulsion (OPE) was used as the control. The emulsion type was identified by observing the emulsion dispersibility in canola oil or water. The emulsion belonged to the oil-in-water (O/W) type if it could disperse in water and aggregate in canola oil. In contrast, the emulsion was water-in-oil (W/O) type [28].

### 2.11. Storage Stability

The 8 mL of OPE and OGPE were stored in transparent glass bottles at 4 °C for one month to evaluate their storage stability. The emulsified phase volume fraction (EPVF) and stability index (SI) of the emulsion were acquired according to the following formulas.

(2) $$EPVF=\frac{He}{Ht}\times 100\%$$

(3) $$SI=\frac{EPVF\;after\;one-month}{EPVF-freshly}\times 100\%$$

where He and Ht are the emulsified phase height and the total emulsion height, respectively.

### 2.12. Statistical Analysis

Each experiment was conducted for at least three repetitions. The significant difference was performed using SPSS 22.0. The data were analyzed through analysis of variance (ANOVA) via Duncan’s multiple comparison test with *p* < 0.05.

## 3. Results and Discussion

### 3.1. Formation of Ovotransferrin Fibrils

**Figure 1:**
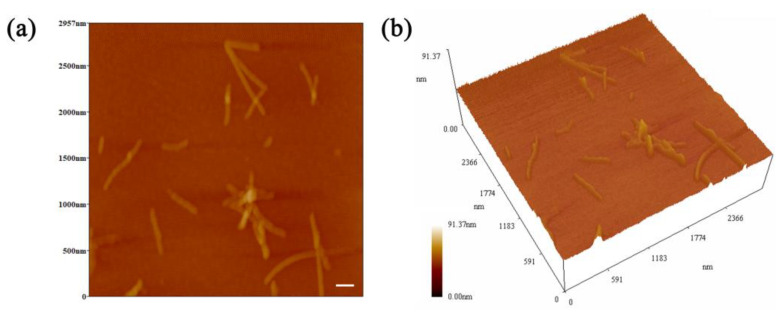
(**a**) Two-dimensional atomic force microscopy (AFM) image of ovotransferrin fibrils (OVTFs). (**b**) Three-dimensional AFM image of OVTFs. The scale bar is 200 nm.

As a way to expand and enrich the functional properties of proteins, protein fibrillation is usually realized via the self-assembly of peptides [29,30]. The self-assembly of spherical proteins into fibrils requires the conditions of acid–heat and appropriate ionic strength [31]. Therefore, ovotransferrin fibrils (OVTFs) were obtained in this study by heating the OVT solution (pH 2.0, 100 mM NaCl) for 24 h at 90 °C. Figure 1 shows the morphology of OVTFs through atomic force microscopy (AFM). Straight fibrils were dispersed on the mica sheet, which demonstrated the successful fabrication of OVTFs. Subsequently, OVTFs were further combined with gum arabic (GA) to explore the interactions between protein fibrils and polysaccharides and the effects on emulsion.

### 3.2. Turbidity of OVTF–GA Complexes

**Figure 2:**
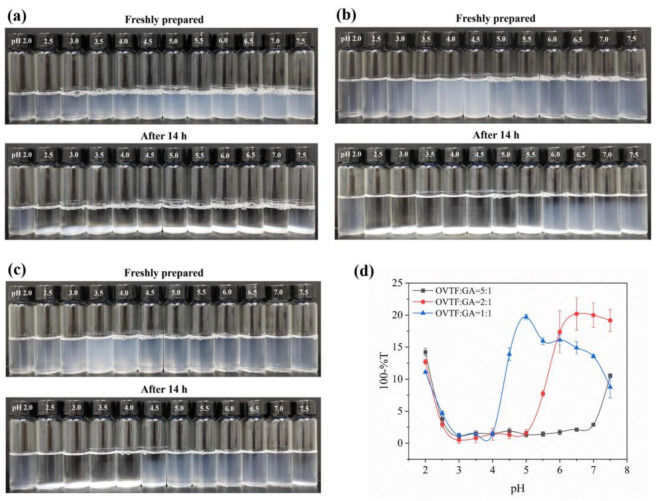
Effect of mass ratios of OVTF to gum Arabic (GA) ((**a**), 5:1; (**b**), 2:1; (**c**), 1:1) and pH on visual appearance of OVTF–GA complexes at room temperature (Top: freshly prepared; Bottom: after 14 h of storage). (**d**) Turbidity of the upper portion of the solution after storage of complexes with different OVTF/GA mass ratios ((**a**), 5:1; (**b**), 2:1; (**c**), 1:1) at different pH values.

Turbidity is an intuitive way to characterize the degree of aggregation (number and size of aggregates) and stability of particles in a system. The higher the degree of particle aggregation, the greater the turbidity will be. The formation and characteristics of OVTF–GA complexes can be significantly influenced by the pH and the ratio of OVTFs and GA. The pH can affect the charge of OVTFs and the ionization degree of GA. When the pH is below the isoelectric point (pI), the OVTFs are positively charged and combine with the negatively charged GA through electrostatic interactions. Furthermore, the amounts of polysaccharides added may affect the stability of complexes. A small number of polysaccharides may not be sufficient to cover the surface of the protein fibrils, resulting in the precipitation of complexes. Therefore, OVTF–GA complexes were fabricated at different pH values (2.0–7.5) and mass ratios (OVTF:GA = 5:1, 2:1 and 1:1). The visual appearances of the OVTF–GA complexes with different pH values and mass ratios before and after storage at room temperature are observed in Figure 2. Overall, the OVTF–GA complexes could be stabilized over a wider pH range as the GA ratio increases (Figure 2a–c). This may be due to the fact that more GA present in the solution binds to the OVTFs and covers the surface area of the OVTFs more comprehensively, which improves the steric hindrance of OVTFs, avoiding aggregation and precipitation. At pH 2.0, the ratio of OVTFs to GA did not have a significant effect on turbidity, and the OVTF–GA complexes showed high turbidity values at all ratios. It is speculated that the possible explanation is the protonation of the carboxyl groups in GA at pH 2.0, which is less charged, resulting in weaker electrostatic interactions with OVTFs, allowing the OVTF–GA complexes to carry more charge and remain stable through electrostatic repulsion. In contrast, the turbidity values of OVTF–GA complexes with different mass ratios were close to zero between pH 3.0 and 4.0, with the possible explanation being that flocculation of OVTF–GA complexes occurred and the settling during the storage time led to a decrease in turbidity. It is also noteworthy that the turbidity of the complexes formed at an OVTF/GA of 2:1 was the greatest at pH 7.5, much higher than at 5:1 and 1:1. It is speculated that there may be two reasons for this phenomenon: (I) Although the OVTFs are negatively charged at pH 7.5, some of the cationic plaques are present [32]. Therefore, weak electrostatic interaction may exist between the OVTFs and GA. (II) At pH 7.5, the hydrogen bonding may become the main force to promote the formation of OVTF–GA complexes. Therefore, the GA can combine with the OVTFs, and the complexes reach maximum turbidity at 2:1, which may be due to the strongest hydrogen bond interaction. Summarized in conjunction with Figure 2, the complex suspensions of OVTFs with GA at a mass ratio of 1:1 showed more pronounced turbidity and excellent stability after storage at pH around 5.0.

**Figure 3:**
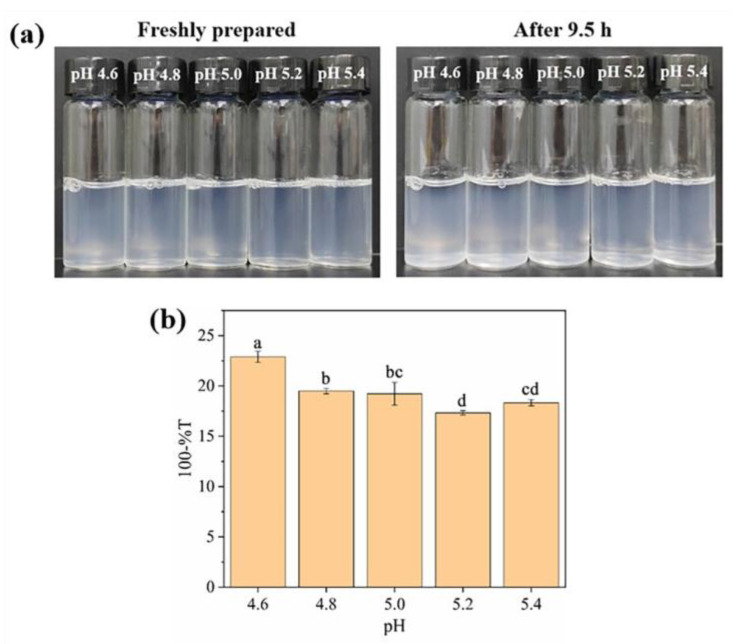
Effect of pH on visual appearance (**a**) and turbidity (**b**) of OVTF–GA complexes with a mass ratio of 1:1 at room temperature. Different letters show significant differences (*p* < 0.05).

Accordingly, OVTF–GA complexes with a 1:1 mass ratio of OVTFs to GA at pH near 5.0 were further discussed. As shown in Figure 3, dispersive stability observations and turbidity measurements of OVTF–GA complexes were carried out in the pH range of 4.6–5.4. It was observed that a more stable and turbid suspension of the complex was formed at pH 4.6. This is because pH 4.6 is further away from the pI of OVTFs in the range of pH 4.6–5.4, which makes the fibrils more positively charged. Stronger electrostatic interactions between OVTFs and GA resulted in a stable complex.

### 3.3. ζ-Potential

**Figure 4:**
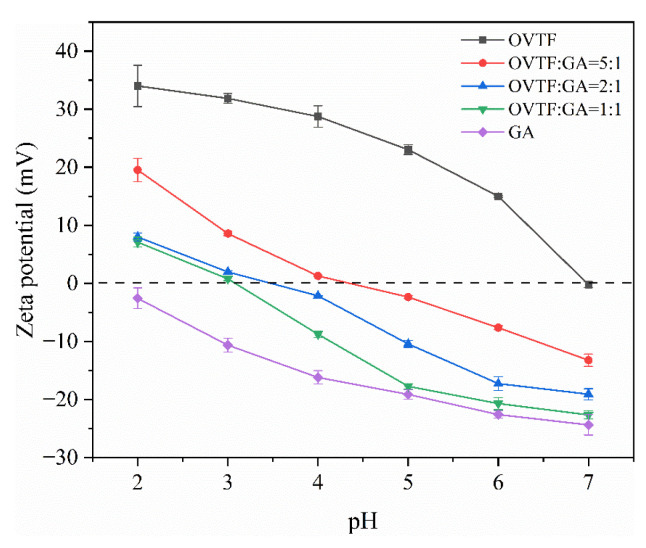
ζ-potential of OVTF, GA and OVTF–GA complexes at different pH values.

The complexation between OVTFs and GA is investigated by measuring the ζ-potential. Figure 4 shows the ζ-potentials of different samples. As displayed in Figure 4, as the pH augmented from 2.0 to 7.0, the positive charge carried by OVTFs gradually decreased due to the approach of the isoelectric point of OVTFs. The ζ-potential of GA reduced from −2.57 mV to −24.37 mV with the increase in pH. The result of ζ-potential exhibited that the OVTFs and GA carried opposite charges in the range of pH 2.0 to 7.0, which was profitable to the formation of OVTF–GA complexes. The charge carried by the OVTF–GA complexes changed from a positive value to negative value when pH was less than pI of OVTFs, suggesting that the combination of the negatively charged GA and the positively charged OVTFs through electrostatic attraction. When the ζ-potential of the OVTF–GA complexes was zero, it meant that charge neutralization occurred between OVTFs and GA. In addition, the ζ-potential of OVTF–GA complexes reduced with the increase in pH and the decrease in OVTF/GA ratio. When the mass ratio of OVTFs to GA was 1:1, complexes exhibited more charge. As shown in Figure 4, it can be seen that the change in the trend of the ζ-potential of the complexes is closer to GA at different ratios of OVTFs and GA. Since ζ-potential is a surface property of particles, when polysaccharides are adsorbed on the fibril surface, the ζ-potential nature of the complexes is closer to GA. Moreover, although GA carried less negative charge at pH 2.0, the ζ-potential of OVTF–GA complexes at different OVTF/GA ratios decreased very significantly. This may be related to the structure of fibrils and polysaccharides. OVTFs are formed by self-assembly of polypeptides with a dense structure, while polysaccharides exhibit a loose linear structure. With a high density of fibrils and a low density of polysaccharides, more polysaccharides will be bound to the fibrils at the same mass ratio, resulting in a significant decrease in their ζ-potentials. Higher surface charges generally lead to excellent stability. Therefore, based on the above analysis, the optimal mass ratio of OVTFs to GA and pH to prepare OVTF–GA complexes were 1:1 and 4.6, respectively.

### 3.4. Morphology Observation

**Figure 5:**
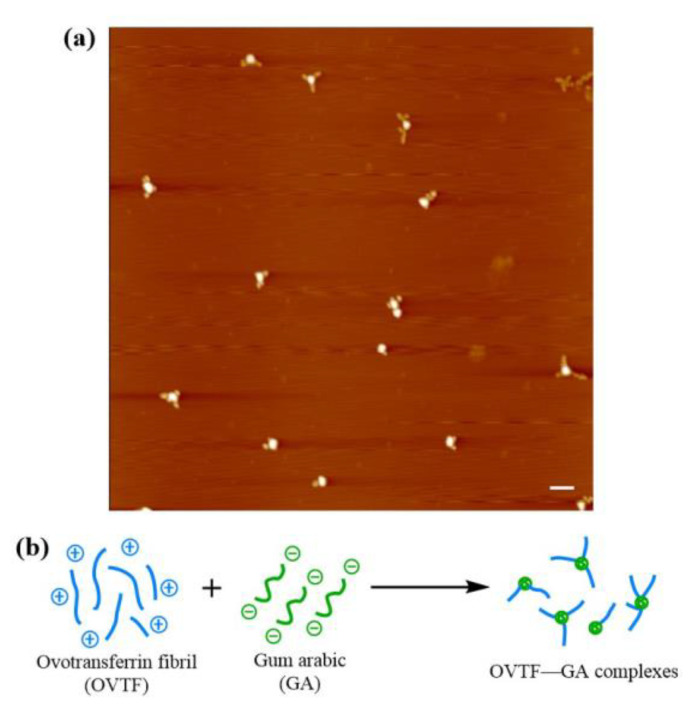
AFM image (**a**) and schematic diagram of the formation mechanism (**b**) of OVTF–GA complexes (1:1 ratio, pH 4.6). The scale bar is 500 nm.

The morphology of OVTF–GA complexes at optimal preparation conditions was characterized by AFM. OVTF–GA complexes exhibited the knot-like structure shown in Figure 5a, which might be due to the following reasons. GA is composed of arabinogalactan (AG), arabinogalactan protein (AGP), and glycoprotein (GP). The binding of polysaccharides to proteins in AGP is via hydrophilic carbohydrate chimeras attached to hydrophobic polypeptide chains, exhibiting a wattle blossom structure. There are a few polypeptides in the OVTFs dispersion, which may be due to the fact that the formation of OVTFs undergoes the process of protein hydrolysis and self-assembly. OVT peptides exhibit hydrophobicity, which is similar to the hydrophobic polypeptide chains of AGP. In the mixing process of OVTFs and GA, a part of AG might combine with polypeptides, resulting in the formation of a wattle blossom structure. Driven by electrostatic attraction, adjacent OVTFs and wattle blossom-like GA formed OVTF–GA complexes, as depicted in Figure 5a. The formation mechanism of OVTF–GA complexes is illustrated in Figure 5b.

### 3.5. Contact Angle(θ)

**Figure 6:**
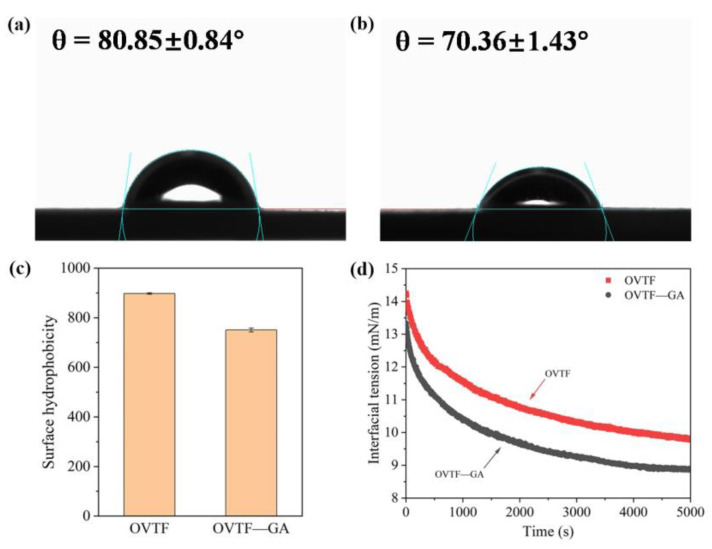
Contact angle (θ) of OVTF (**a**) and OVTF–GA complexes (**b**). Surface hydrophobicity (**c**) and dynamic interfacial tension (**d**) of OVTF and OVTF–GA complexes.

Solid particles can be adsorbed on the oil–water interface of emulsions when they can be partially wetted by both the water and the oil [33]. As illustrated in Figure 6a,b, θ values of OVTFs and OVTF–GA complexes were 80.85° and 70.36°, respectively. The results showed that OVTFs and OVTF–GA complexes with outstanding wettability could be partially wetted by oil and water phases and were suitable to stabilize Pickering emulsions. In addition, θ values are related to the type of Pickering emulsions. When θ is less than 90°, oil-in-water (O/W) emulsion is easily prepared. Water-in-oil (W/O) Pickering emulsion is usually obtained when θ is greater than 90° [34]. Therefore, OVTFs and OVTF–GA complexes were profitable for the stabilization of O/W Pickering emulsions. Furthermore, the θ of OVTF–GA complexes was lower than OVTFs, suggesting that OVTF–GA complexes showed lower surface hydrophobicity, which was due to the binding of hydrophilic GA to OVTFs.

### 3.6. Surface Hydrophobicity

The surface hydrophobicity of different samples was compared. It can be seen from Figure 6c that the addition of GA resulted in the reduction in the surface hydrophobicity of OVTFs. The surface of the OVTF–GA complexes was more hydrophilic than that of the OVTFs. On the one hand, GA is a molecule with strong hydrophilicity, and a large number of GA molecules interact with OVTFs, introducing more hydrophilic groups. On the other hand, the adsorption of GA on OVTFs covers some exposed hydrophobic regions on the surface of OVTFs and reduces the hydrophobic site on the surface of OVTFs for binding to hydrophobic fluorescent probe, thereby reducing the surface hydrophobicity of OVTFs.

### 3.7. Dynamic Interfacial Tension

As shown in Figure 6d, the interfacial tension of OVTF and OVTF–GA complexes reduced with the increase in time. Moreover, the dynamic interfacial tension of different particles decreased rapidly and then slowly, which was related to the adsorption process of the particles at the interface. The process of particle adsorption at the interface can be divided into three steps: diffusion, adsorption and rearrangement. In the first stage, the particles diffuse rapidly from the aqueous phase to the oil–water interface, resulting in a rapid reduction in interfacial tension. After that, the interfacial tension decreases slowly because the particles are saturated at the interface, and further conformational rearrangement occurs [35,36]. OVTF–GA complexes showed a lower interfacial tension than OVTFs, indicating that OVTF–GA complexes had a stronger ability to reduce the interfacial tension. As shown in Figure 6d, the OVTF–GA complexes could reduce the interfacial tension more rapidly than the OVTFs, which demonstrated that the complexes could be adsorbed to the interface more quickly. As exhibited in Figure 4, OVTF–GA complexes had less net charge, resulting in the lower electrostatic repulsion on the interface, which was profitable to the absorption of OVTF–GA complexes. Generally, the decrease in the interfacial tension of complexes is associated with increased emulsifying ability and emulsion stability. Therefore, OVTF–GA complexes as stabilizers may have better emulsification ability and can improve the stability of Pickering emulsions.

### 3.8. Emulsifying Ability

Polysaccharides show many potential applications in food industries [37]. For example, the addition of polysaccharides is a feasible method to modify protein fibrils. The interaction between polysaccharides and protein fibrils can improve the physicochemical characteristics of protein fibrils, thereby showing functional properties different from those of protein fibrils and polysaccharides. Complexes based on protein fibrils and polysaccharides not only show potential as delivery systems for bioactive ingredients [11,38,39] but also have excellent emulsification properties [40,41].

The OVTF–GA complexes were used as emulsifiers to construct the oleogel-based Pickering emulsion to evaluate its emulsifying capacity. As a new type of delivery system, oleogel-based Pickering emulsion can further improve the stability of the system and the water dispersibility of bioactive substances, which has a broad application prospect in food industries [42,43]. In our previous work, oleogel composed of canola oil and candelilla wax, was fabricated [16]. The concentration of candelilla wax was controlled at 1.1% (*w*/*w*) to promote the formation of oleogel. In the current research, OVTF-stabilized oleogel-based Pickering emulsion (OPE) and OVTF–GA complex-stabilized oleogel-based Pickering emulsion (OGPE) were prepared in order to better explore the ability of OVTFs and OVTF–GA complexes to stabilize Pickering emulsion.

**Figure 7:**
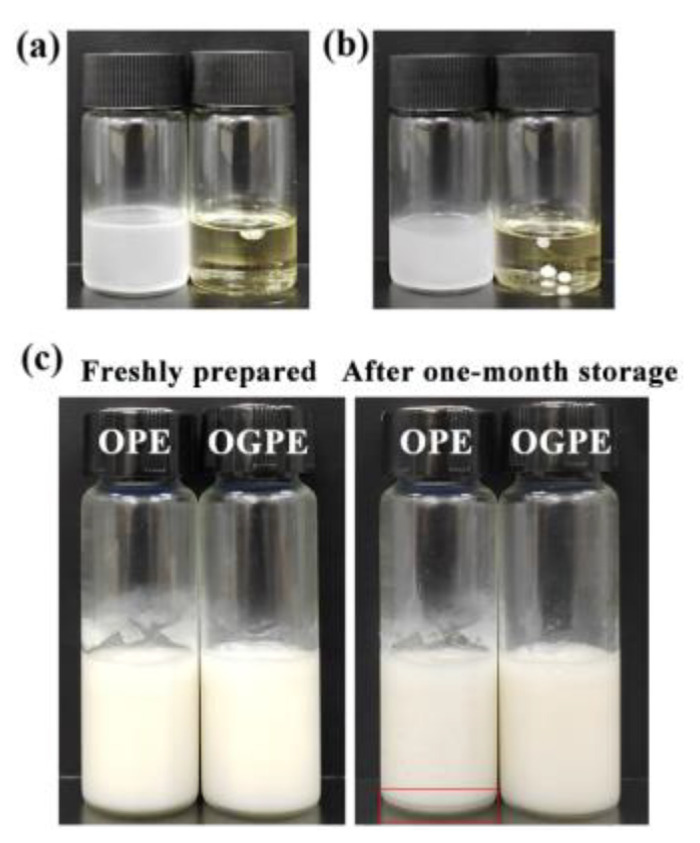
Type identification of OVTF-stabilized oleogel-based Pickering emulsion (OPE, (**a**)) and OVTF–GA complex-stabilized oleogel-based Pickering emulsion (OGPE, (**b**)). (**c**) Visual observation of OPE and OGPE before and after storage.

**Figure 8:**
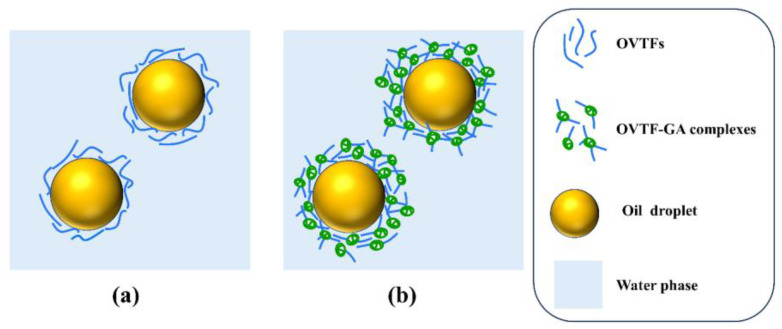
Schematic diagram of possible stabilization mechanism of OPE (**a**) and OGPE (**b**).

Since OPE and OGPE could be dispersed in water and exhibited aggregation in canola oil, both emulsions belonged to O/W type emulsions, suggesting that OVTFs and OVTF–GA complexes had better wettability to water than to oil, which further confirmed the results of θ (Figure 7a,b). Figure 7c depicts the visual appearance of OPE and OGPE before and after storage. After one-month storage, emulsion stratification was noticed in OPE, whereas OGPE did not show obvious stratification. In addition, Table 1 displays the emulsified phase volume fraction and the stability index of OPE and OGPE before and after one-month storage. The results indicated that both emulsions exhibited long-term storage stability with a stability index above 97%. OVTF–GA complexes showed excellent emulsification characteristics, which might be due to the following reasons. First, compared with OVTFs, OVTF–GA complexes containing polysaccharides as stabilizers for OGPE could resist deformation of the droplet structure, which might be due to the improvement of the interface thickness and integrity (Figure 8). In addition, through the direct observation of the OGPE oscillation experiment, it was found that OGPE had high viscosity and gelatinous structure. The high viscosity was beneficial to slowing down the movement of emulsion droplets, resulting in improved emulsion stability.

## 4. Conclusions

This research investigated the formation process of ovotransferrin fibril–gum arabic (OVTF–GA) complexes, as well as the driving force, structure, and emulsification properties. Stable OVTF–GA complexes were obtained when the mass ratio of OVTFs to GA and pH were 1:1 and 4.6. It was observed morphologically that GA stacked as spherical clusters wrapped around the OVTFs. This may be due to the fact that the arabinogalactan (AG) portion of GA bound to the peptide produced via hydrolysis to form a wattle blossom structure, which later bound to the adjacent OVTFs to form a specific knot-like structure. The electrostatic interactions were speculated as being the main driving force for the formation of OVTF–GA complexes based on the results of turbidity and ζ-potential. The electrostatic interactions as the main driving force for the formation of OVTF–GA complexes were confirmed. The incorporation of GA reduced the surface hydrophobicity of OVTFs. The OVTF–GA complex-stabilized oleogel-based Pickering emulsion (OGPE) had long-term storage stability. The results of this study will help to construct and apply protein fibril–polysaccharide complexes according to specific needs, which will lead to functional value-added components with desirable properties and provide natural emulsifiers with excellent emulsification properties for the food industry.

## Figures and Tables

**Table 1:** Emulsified phase volume fraction and stability index of OVTF-stabilized oleogel-based Pickering emulsion (OPE) and OVTF–GA complex-stabilized oleogel-based Pickering emulsion (OGPE).

Emulsion	Emulsified Phase Volume Fraction (%)		Stability Index (%)
	Fresh	After One-Month Storage	
OPE	98.3 ± 0.2 ^a^	95.7 ± 0.9 ^a^	97.3 ± 0.1 ^a^
OGPE	100.0 ± 0.0 ^b^	99.4 ± 1.1 ^b^	99.4 ± 1.1 ^b^

Note: Different letters within the same column shows significant differences (*p* < 0.05).

## Data Availability

The data herein presented are available upon request from the corresponding author.
